# Adnexal Torsion in Pediatric Age: Does Bolli's Score Work? Report of Two Cases

**DOI:** 10.1155/2019/9701874

**Published:** 2019-12-08

**Authors:** L. Giambanco, V. Iannone, M. Borriello, G. Scibilia, G. Sozzi, V. Chiantera, P. Scollo

**Affiliations:** ^1^Ob & Gyn Department, S. Antonio Abate Hospital, Trapani, Italy; ^2^Ob & Gyn Department, Cannizzaro Hospital, Catania, Italy; ^3^Oncol Gyn Department, Civico Hospital, Palermo, Italy

## Abstract

Adnexal torsion is a surgical emergency requiring early diagnosis in order to avoid demolitive surgery. Adnexal torsion's diagnosis could be very difficult in pediatric patients because children cannot explain symptoms accurately. Furthermore reproductive organs lie high in abdomen, causing unclear examinations findings. For reducing diagnostic mistakes or delay clinical and hematological criteria could be useful. No radiological criteria (CT or MRI) should be taken in count because of the costs and the required time. By combining clinical presentation in patients with OT three useful diagnostic variables have been identified: age, duration of pain, vomiting. Presence of vomiting, short duration of abdominal pain and high CRP levels have great predictive value for the diagnosis of adnexal torsion. In those patients an exploratory laparoscopy should be performed without any doubt and/or delay. These data may aid physicians in the evaluation of abdominal pain in premenarchal girls.

## 1. Introduction

Adnexal torsion represents a surgical emergency happening both in premenarchal and postmenarchal age. According to many observational studies, in premenarchal age ovarian torsion often occurs in healthy ovaries, without any evidence of ovarian cysts [1, 2].

Adnexal torsion is an emergency that need prompt diagnosis and timely surgical intervention and detorsion in order to avoid ovarian and tubal damage. However signs and symptoms of adnexal torsion are aspecific and could mimic other surgical emergencies [3].

Clinical presentation is aspecific, with lower abdominal pain uni- or bi-lateral, diasappearing after 12 hours. Vomiting and fever could be present. There is no serum biological marker of adnexal torsion, even if elevated C-reactive protein (CRP) is a common finding.

The longer the diagnosis is delayed, the lower is the chance of preserving ovarian function. The twisting of infundibolopelvic and uterovaric ligaments could lead to temporary occlusion of ovarian vessels and ovarian damage that may or may not be reversible. Diagnosis is confirmed during laparoscopy, in the meanwhile surgeons would consider the better surgical option: radical or conservative. An animal study showed that ovarian necrosis develops after almost 36 hours of torsion and occlusion of ovarian vessels [2, 4]. Conservative surgery detorsioning the adnexa does not seem to worsen postoperative course; it's proven that ovaries still work even though macroscopically they appear blueish and oedematous.

The treatment of ovarian torsion (OT) is often delayed because of diagnostic uncertainty and dependence on radiologic confirmation. In contrast, when testicular torsion (TT) is suspected, diagnosis and management are expedited despite lack of certainty, and operative exploration is not delayed [5].

Both pediatric and gynecological surgeons should be aware that adnexal torsion could happen in premenarchal age, without any ovarian mass as primum movens. Diagnostic suspicion warrants a prompt laparoscopy for confirming diagnosis. Laparoscopy represents the gold standard surgical approach.

## 2. Case Report 1

An infant of 19 months was referred to our department from a primary care service because of abdominal pain, which appeared three days before, vomiting and fever. She had no special medical history and other symptoms except for poor oral intake previous days. Abdominal pain and vomiting stopped after 24 hours. Fever was not so high, between 37.5 and 37.9°C. There was no leukocytosis, bowel was normal functioning. CRP was elevated (32 mg/l). Abdominal palpation caused pain. Ultrasound evaluation revealed an enlarged mass in left pelvic area. Thus, we planned an emergent laparoscopy for diagnostic confirmation and surgical treatment.

**Figure 1:**
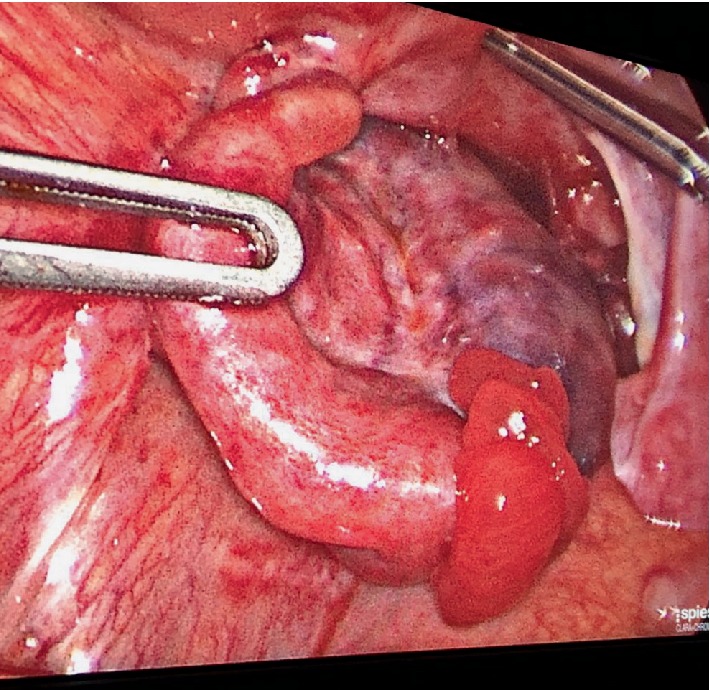
Left adenaxal torsion in 2 years old baby, >36 hours symptoms' start.

At the time of laparoscopy left adnexa was twisted on its axis, blackwish, bloated (Figure 1). Left adnexectomy was performed because time presentation lasted more than 3 days and intraoperative macroscopic findings were suggestive for massive necrosis. Postoperative course was uneventful and baby girl was discharged after 48 hours. Pathologist confirmed extended necrosis and hemorrhagic infarction in left ovary and salpinx.

## 3. Case Report 2

**Figure 2:**
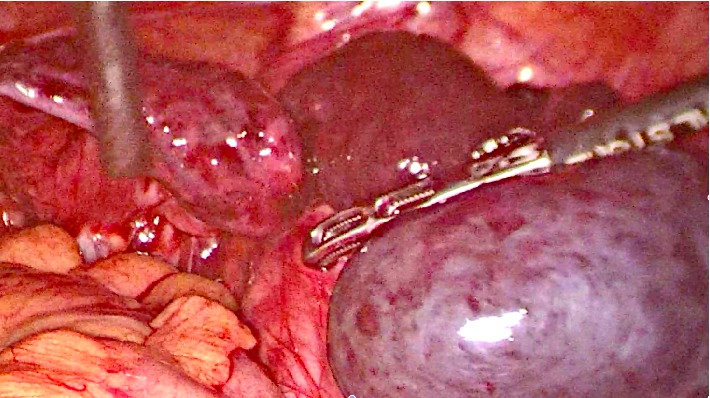
Right adnexal torsion in 10 years old children: <12 hours pain.

**Figure 3:**
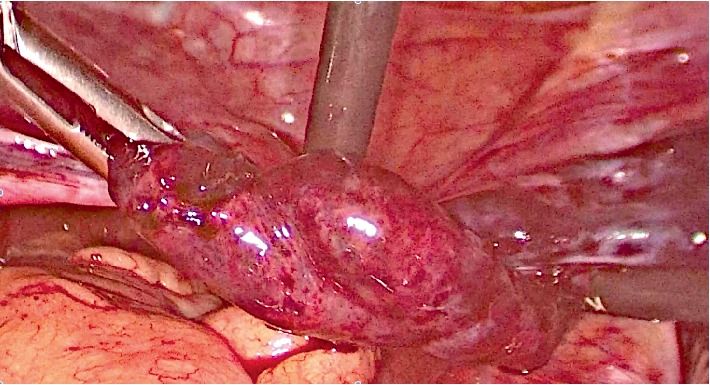
Left adnexal torsion, same patient.

**Figure 4:**
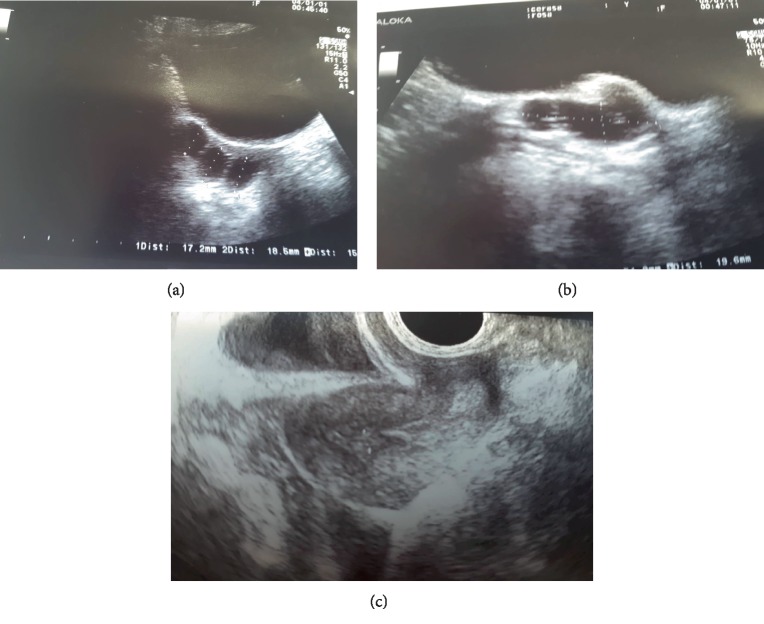
(a), (b), (c) Pelvic scan with TA probe after 6 months, patient n2, sactosalpinx.

A 10 years old girl went to emergency service of our hospital for abdominal pain and vomiting started 10 hours before. After surgical consultation, gynaecologists suspected ovarian torsion. Transabdominal pelvic scan showed bilaterally enlarged ovaries, free fluid in the pouch of Douglas, CRP level was 14 mg/l. We decided to perform laparoscopy: intraoperative findings showed syncronous bilateral adnexal torsion with multiple twists. On the right side (Figure 2) serosa was blueish but after detorsion became reddish and the ipsilateral ovarian volume became regular. On the left side, unfortunately, ovary was enlarged with infarction and necrosis (Figure 3). Thus we decided to perform conservative surgery, for right ovary and salpinx. Left adnexum was removed with classical technique: bipolar forceps and monopolar scissors. Postoperative course was uneventful and the girl was discharged after three days. Transabdominal pelvic scan one month later showed normal ovarian structure and no tubal anomalies. Six months later, the girl came back to the emergency unit for acute pelvic pain, ultrasound evaluation showed an irregular sactosalpinx in right iliac fossa (Figures 4(a)–4(c)).

## 4. Discussion

Adnexal torsion in premenarchal patients occurs rarely, its symptoms are similar to older patients, but involves a normal adnexa in 69% of cases [6]. Compared with older women, premenarchal patients with adnexal torsion are more commonly found with normal ovarian volume [1, 2, 7].

Mechanism of torsion of normal ovaries remain unclear. In premenarchal girls utero-ovarian ligaments are normally elongated. Hypermobility due to an elongated utero-ovarian ligament and hyperlaxity of mesosalpinx could represent risk factors for torsion [8].

Adnexal torsion's diagnosis could be a diagnsotic challenge in pediatric age because children cannot explain symptoms accurately, furthermore reproductive organs lie high in abdomen, causing unclear examinations findings [9].

In comparison to boys with testicular torsion, girls with suspected OT waited 2.5 times as long for diagnostic imaging and 2.7 times as long to be taken to the operating room [5]. In addition, gonadal salvage rate is significantly worse for girls compared with boys with TT. More urgent intervention for OT, with liberal use of diagnostic laparoscopy and without reliance on a definitive diagnosis by imaging, should be considered in girls with lower abdominal pain.

For reducing diagnostic mistakes or delay some clinical and/or hematological criteria could be useful. CT or MRI should be avoided because of the costs and the required time. Bolli et al. [10], by combining clinical presentation in patients with OT, identified 3 useful diagnostic variables: age, duration of pain, vomiting. Leukocytosis and enlarged ovaries resulted unuseful for early ovarian torsion diagnosis. Meanwhile the parameter elevated PRC became significant [9]. CRP levels usually rise up in response to inflammation and tissue necrosis [10]. In case of adnexal torsion CRP high concentration could be explained by the process of tissue damage in ovarian torsion compared to acute adnexal pathologies [11].

The Authors established an ovarian torsion score: age (points: number of years), minus 3 points (if vomiting was yes), plus 1 point (if pain duration was above 12 hours). The lesser the points, the more likely the girl suffered ovarian torsion. The cut off value to identify girls with ovarian torsion and girls with ovarian cysts was 11.5 points. In a subgroup analysis of the Bolli's score in girls aged 2–12 years the AUC was 0.94, sensitivity of positive ovarian torsion rose up to 1.00 [10]. The variables “vomiting” and “pain duration” were predictive for ovarian torsion.

Bolli's score would have well worked for both case reports. In the first case, baby girl aged 19 months (1 point), absence of vomit (0 point), pain lasting more than 12 hours (1 point), final Bolli's score was 2.

For 10 years old girl, with syncronous bilateral adnexal torsion, Bolli' score reached 7 (age 10, minus 3 point for vomiting, 0 points for pain (lasting less than 12 hours). The variables “vomiting” and “pain duration” were highly predictive for ovarian torsion [10]. Vomiting usually matches with the onset of abdominal pain, probably due to vagal reflex. Pain duration in girls with adnexal torsion is shorter than in patients with ovarian cysts [10].

## 5. Conclusions

Diagnosis of pediatric adnexal torsion is difficult and often delayed, even if it represents from 20% to 30% of surgical pediatric emergencies [11]. Pain and tenderness may not be isolated to a unilateral lower quadrant. Although traditionally considered a postmenarchal problem, in a pediatric academic emergency department adnexal torsion occurred with similar frequency in premenarchal and postmenarchal girls. Infants with an ongoing adnexal torsion present with feeding intolerance, vomiting, abdominal pain and distension [12]. The presence of vomiting, short duration of abdominal pain and high CRP levels has a great predictive value for the diagnosis of adnexal torsion. In those patients an exploratory laparoscopy should be performed promptly [10].

The potential for organ salvage means that adnexal torsion should be considered in all females presenting with acute abdominal pain regardless of age or menstrual history [13, 14]. A study on animals showed that necrosis starts developing after occlusion of ovarian vessels for 36 hours or longer [15]. Nowadays there is no scientific evidence that detorsion of twisted ovary increases adverse events, postoperatively [16]. Nonetheless patients with an ovary apparently necrotic (black color, loss of normal anatomic structure) during intraoperative evaluation should undergo salpingo-oophorectomy [7].

Bolli's score [10] could represent a relevant dagnostic tool for early diagnosis or suspicion of adnexal torsion. It could be applied to girls aged 2–14 years with great sensitivity and PPV. It doesnot need radiological help, just clinical data. Blood test, such increased PRC could confirm diagnostic suspect. Pelvic ultrasound could be used as first-line imaging study for premenarchal girls with abdominal pain with suspected adnexal torsion. Ultrasound is not expensive, it is in every emergency area and radiological risk free. Pelvic magnetic resonance and/or computed tomography scan shoud be avoided: they are expensive, time requiring and have similar diagnostic value to ultrasound study [17]. It's not useful analyzing specific sonographic characteristics (echotexture, peripheral cysts, flow on Doppler). According to Servaes, 60% of proven adnexal torsion had normal blood flow assessed on Doppler sonography [18]. Thus it is better to focus on ovarian volume enlarged or not).

These data may aid the physician in the evaluation of abdominal pain in premenarchal girls [19] and avoid wasting diagnostic time that could result in ovarian damage and demolitive surgery.
